# Use and perceived usefulness of a just-in-time resonance breathing intervention adjunct for substance use disorder: Contextual and physiological predictors

**DOI:** 10.3389/fpsyt.2022.945751

**Published:** 2022-09-07

**Authors:** Julianne L. Price, Marsha E. Bates, Anthony P. Pawlak, Sarah Grace Uhouse, Sabrina M. Todaro, Julie Morgano, Jennifer F. Buckman

**Affiliations:** ^1^Cardiac Neuroscience Laboratory, Center of Alcohol and Substance Use Studies, Rutgers University—New Brunswick, Piscataway, NJ, United States; ^2^Department of Kinesiology and Health, Rutgers University—New Brunswick, Piscataway, NJ, United States; ^3^Department of Psychology, Rutgers University—New Brunswick, Piscataway, NJ, United States; ^4^Department of Psychology, College of Health Sciences, University of Rhode Island, South Kingstown, RI, United States

**Keywords:** heart rate variability, baroreflex, resonance breathing, substance use disorder, clinical trial, cardiovascular, craving, just-in-time intervention

## Abstract

Craving for alcohol and other drugs is often described as a momentary hyperarousal state that interferes with one's ability to use top-down strategies. As such, it may be best interrupted ‘in the moment' through bottom-up modulation. We recently reported that episodic resonance paced breathing (eRPB) delivered via mobile phone app as an add-on to outpatient treatment for substance use disorder (SUD) was effective at dampening craving over the course of an 8-week intervention (NCT#02579317). However, not all participants engaged with the eRPB app and there was high intra- and inter-individual variability in weekly ratings of usefulness. Here we examined baseline demographic, physiological, and psychiatric measures as well as time-varying exposure to positive, negative, and temptation craving triggers as predictors of frequency of eRPB app use and ratings of usefulness. Seventy-seven outpatient women were randomized to an eRPB (0.1 Hz) or a faster paced breathing sham (0.23 Hz) condition. Baseline measures were assessed within the first 3 weeks of treatment entry prior to randomization. App use frequency, ratings of usefulness, and trigger exposure were measured weekly throughout the intervention. Variables were entered into marginal means models with forward stepwise model selection and examined as predictors of use and usefulness. Frequent app use was associated with a lifetime alcohol use disorder (AUD) diagnosis (*p* = 0.026), higher ratings of usefulness (*p* < 0.001), and fewer exposures to positive triggers (e.g., celebration, socialization; *p* < 0.001). There was a trend-level association between frequency of app use and greater cardiovascular capacity at baseline (*p* = 0.088). Higher ratings of usefulness were associated with greater exposure to negative triggers (e.g,. loneliness, frustration; *p* < 0.001) and parasympathetic dysregulation at baseline (*p* = 0.05). A positive relationship between app use frequency and ratings of usefulness was present only in the eRPB group (*p* = 0.045). Matching ideal candidates and moments to an arousal modulation anti-craving intervention can help streamline screening and implementation of eRPB in the treatment of SUD.

**Clinical Trial Registration:**

https://clinicaltrials.gov/ct2/show/NCT02579317, identifier NCT02579317.

## Introduction

Craving for alcohol and other drugs is a core feature of substance use disorder (SUD) and a primary interrupter of recovery (1, 2). The experience of craving is complex and involves intense emotionality, behavioral activation, and autonomic physiological experiences that can block higher order cognitive processes (3–7). Physiologically, craving is marked by a shift toward sympathetic control, including increased heart rate (HR) (8, 9) and blood pressure (9, 10), decreased heart rate variability (HRV) (11, 12), and rapid respiration (13, 14). Bottom-up communication of these physiological correlates of craving can persist for hours (15–17), disengage top-down processes (18–20), and promote unintended substance use (7, 21, 22). A number of pharmacological and psychological anti-craving interventions have received empirical support (23–27). Supplementation by a just-in-time, bio-behavioral intervention that interrupts the relay of visceral craving signals from the body to the brain theoretically could create a temporal window for an individual to recruit top-down processes in risky contexts and thereby enhance existing approaches to support recovery in everyday life (20, 28–32).

HRV biofeedback, a bottom-up intervention used to dampen arousal, slows and paces respiration to drive vagally-mediated HR oscillations (~0.2–0.33 Hz) to match the periodicity of the HR baroreflex (~0.1 Hz) (33–35). In doing so, immediate increases in HRV and baroreflex sensitivity as well as decreases in BP and sometimes HR are observed (34, 36). All of these changes are indicators of parasympathetic control and likely underlie the beneficial effects of HRV biofeedback on stress/arousal (37, 38) and reactivity to appetitive cues (39–41). In addition, HRV biofeedback activates central-autonomic neural pathways that convey cardiovascular information to multiple cortical centers (42); this feedforward path has been proposed to underlie decreased craving for alcohol and other drugs (30, 43–47).

Standard HRV biofeedback is a clinician-guided intervention that requires a trained practitioner to lead a series of breath-based exercises to identify one's unique resonance frequency (i.e., the precise frequency of the periodicity of an individual's HR baroreflex; range: 0.075–0.12 Hz). Individuals are then instructed to practice breathing at their resonance frequency on their own time, usually with the aid of photoplethysmography (PPG) to visually synchronize respiration and HR oscillations; they return weekly for in-person practitioner-guided sessions. The duration of HRV biofeedback involvement, number of in-person sessions, and frequency of self-practice vary, but generally individuals complete weekly in-person visits for 2–10 weeks paired with 5–30 minutes of daily self-practice (45, 48). Self-guided episodic resonance-paced breathing (eRPB) is a parallel, but less intensive intervention strategy that leverages the respiratory strategies of HRV biofeedback. This intervention requires individuals to pace their breath to 0.1 Hz to approximate the resonance frequency of the HR baroreflex (36, 49). Without the assistance of a practitioner, eRPB can be used in the real world through mobile health platforms, allowing the active ingredient of HRV biofeedback to be implemented in daily life.

The utility of a just-in-time intervention like eRPB relies on self-administration of the application. Individual-level factors such as age, general health, mood, and baseline physiological traits have been associated with level of engagement with just-in-time interventions (39, 48, 50); and while racial and ethnic minority individuals find personalized interventions promising (51, 52), societal barriers continue to limit access and decrease enthusiasm for their implementation (53, 54). These studies suggest a need for more research aimed not only at whether a just-in-time intervention is efficacious, but also at quality of implementation, including who uses it, when it is used, and whether it is found to be useful.

The current analyses used data from a randomized clinical trial (RCT) of an adjunctive eRPB intervention for women attending an outpatient behavioral treatment program for SUD. Our initial report on clinical outcomes found improvements in craving that varied with the frequency of eRPB use during intervention weeks in this sample, compared to a breathing sham control group (55). Here, we focused on a complimentary aim of the RCT that addressed when and for whom the eRPB intervention was most useful. We examined a series of *a priori* baseline demographic, substance use, and physiological characteristics as well as time-varying exposure to triggers for substance craving to predict two metrics of utility: frequency of use and self-reported usefulness of eRPB. Based on previous reports that relate age, health, and baseline physiology with use of technology-based interventions, we hypothesized that younger age, greater basal cardiovascular dysregulation, and metrics of poor health would be associated with more frequent use and higher ratings of app usefulness. We further evaluated time-varying predictors of app utility in line with its intended use in daily life. Given that negative emotionality is commonly cited as a trigger to relapse (56, 57), particularly in women (58), it was hypothesized that increased exposure to negative craving triggers (e.g., loneliness, experiencing conflict, feeling shaky) would be associated with greater use and usefulness of an arousal modulating intervention compared to positive affect triggers (e.g., celebration, socialization).

## Materials and methods

### Trial design

The Project IMPACT (In-the-moment Protection Against Craving Triggers; NCT#02579317) design used a parallel-assignment RCT to test whether self-administered, in-the-moment, resonance breathing episodes would improve outcomes for women receiving SUD treatment. Urn randomization was used to assign participants to either the eRPB or sham breathing intervention to maximize the probability of balanced groups with regard to important prognostic characteristics [age 18–30, >30 years; alcohol use disorder (AUD) or other substance use disorder (SUD) diagnosis] and to preserve unpredictability/allocation concealment. The protocol was approved by and conducted in accordance with the Institutional Review Board for the Protection of Human Subjects Involved in Research.

### Recruitment and sample characteristics

Participants were recruited between November 2015 and March 2020 from a community outpatient substance use treatment facility that offered a continuum of care for women. This client-centered facility used evidence-based treatment approaches that optimize clinical care for women and their children including seeking safety (59), motivational interviewing (60), and child parent psychotherapy (61). Consecutive admissions to the program were invited to take part in an 8-week paced breathing study with two arms: 6 breaths per min (eRPB) or 14 breaths per minute (sham breathing control). Inclusion criteria included age between 18 and 65 years, the ability to provide informed consent and complete breathing tasks, and not pregnant. Women who qualified as having a current or lifetime SUD (alcohol included) were included in the study. Six women had achieved more than 30 days of abstinence at the time of enrollment. Women who exhibited severe mental health symptoms did not qualify for the intensive outpatient program; no further psychiatric criteria were applied.

**Figure 1:**
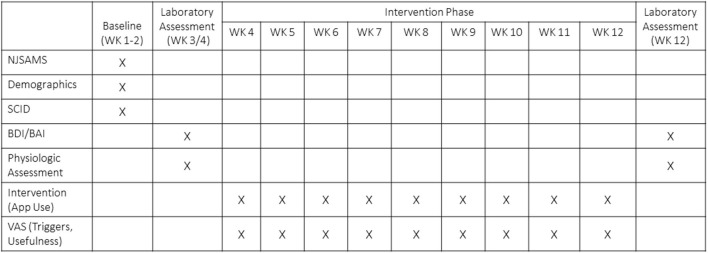
Timeline of study involvement and relevant measures. NJSAMS, New Jersey Substance Abuse Monitoring System; Structured Clinical Interview for DSM-5; BDI, Beck Depressive Inventory; BAI, Beck Anxiety Inventory; VAS, Visual Analog Scale.

A timeline of study involvement and relevant measures can be seen in Figure 1. As part of the intake evaluation, clinicians administered the New Jersey Substance Abuse Monitoring System (NJSAMS), a clinical interview conducted by all NJ state-funded treatment facilities that collects demographic information, substance use history, financial status, medical history, and clinical information to help treatment providers identify the appropriate treatment level for clients. At initial research contact, all participants provided written informed consent (*n* = 107). Participants then completed demographic and health screening information (week 1). During week 2, a trained, graduate-level clinical researcher administered the alcohol and substance use disorder sections of the Structured Clinical Interview for DSM-5 [SCID-5 (62)] to verify diagnoses, the Mini International Neuropsychiatric Interview (MINI) 7.0 (63) to assess psychiatric comorbidities, and the Inventory of Drug Taking Situations [IDTS (64)], which identifies positive, negative, and temptation-related triggers for substance use. The following week, the researcher returned to administer a 90-day TimeLine Follow Back in-person interview [TLFB (65)] that assessed alcohol and other drug use (opiate, stimulant, nicotine, cannabis, hallucinogens); craving scale [Penn Alcohol Craving Scale (PACS) (66)], and depressive and anxiety symptoms inventories [Beck Depressive Inventory, BDI (67) and Beck Anxiety Inventory, BAI (68)]. An in-laboratory session was then scheduled.

**Figure 2:**
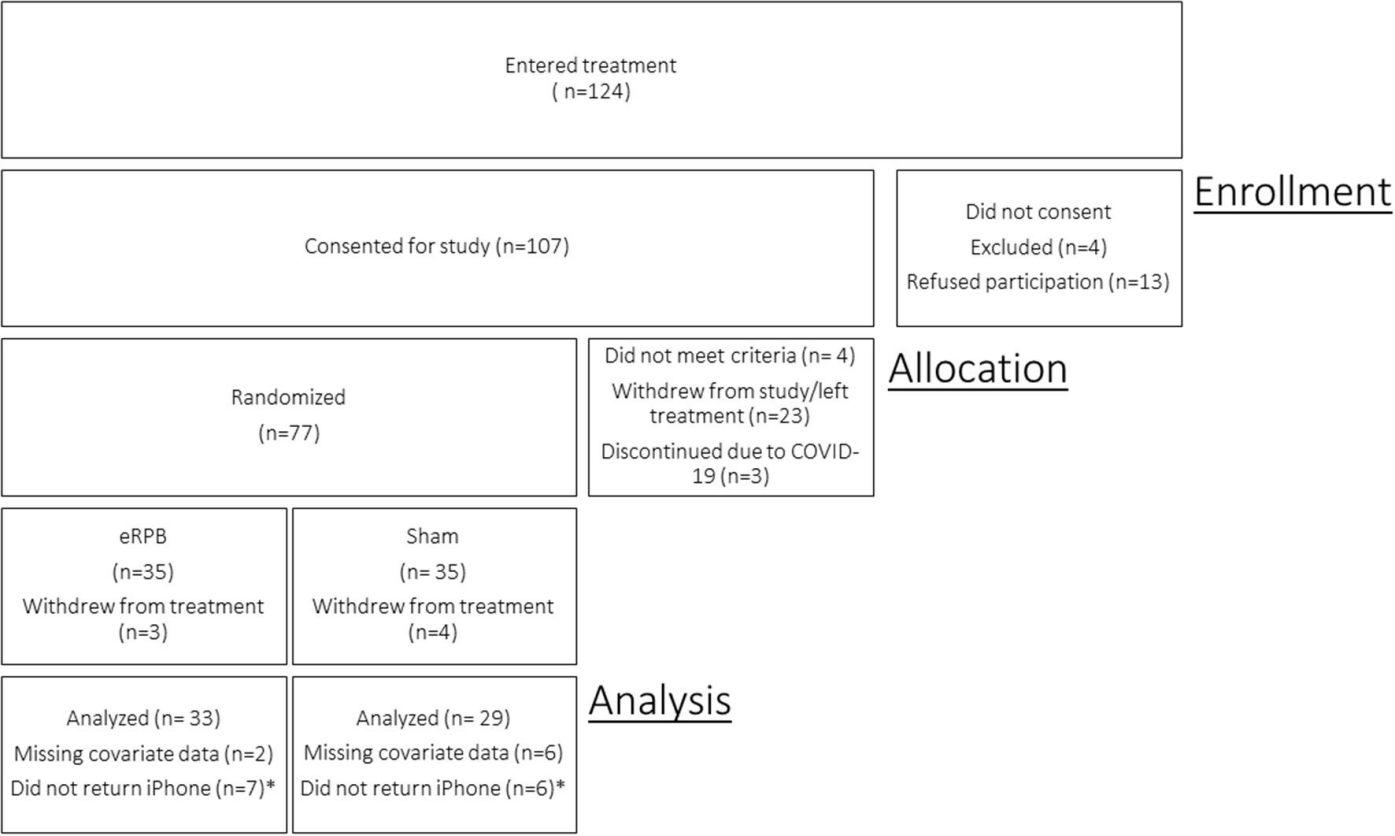
CONSORT enrollment diagram. *Participants remained in the study and reported on usefulness of the intervention but did not return iPhone for app usage data download. Participants are included in model of Usefulness but not Use Frequency.

Of the 107 consented participants, 4 did not meet criteria and were excluded from the study, 17 dropped out of treatment and study procedures prior to the baseline in-lab assessment, and 6 withdrew from study procedures, but remained in treatment. Due to the pandemic-related suspension of non-essential human subjects research in March 2020, three participants were discontinued prior to data collection and study enrollment was terminated before the full target sample could be recruited. Seventy-seven participants completed the baseline in-lab cardiovascular assessment and were randomized. Seven discontinued treatment and study involvement after randomization but before app use data collection. Four participants had unusable baseline cardiovascular data. Time-varying covariate data were missing for four participants. The final sample for the current analyses included 62 participants. Thirteen women reported on the weekly usefulness measure but failed to provide their phone for data uploads and thus are missing app use frequency data. The CONSORT flow diagram is presented in Figure 2.

### Pre-intervention laboratory phase

During the laboratory session, participants were given a light lunch and completed questionnaires and cognitive tasks [Not reported here; see Supplementary material in Price et al. (55)]. Participants then were seated comfortably in front of an LCD TV screen. A standard lead II configuration (arm & ankle) was used for electrocardiogram (ECG) measurement. A cuff sensor for beat-to-beat blood pressure measurement was placed around the second phalange of the right middle finger. A stretch belt with piezoelectric sensors for respiratory measurement was set around the chest. ECG, respiration, and blood pressure were continuously acquired at a 2,000 Hz sampling rate using PowerLab Acquisition System (ADInstruments, Colorado Springs, CO) and Finometer MIDI (Finapres Medical Systems, Enschede, Netherlands). Data were post-processed prior to analysis using WinCPRS software (Absolut Aliens Oy, Turku, Finland) to manually modify artifacts and missed or irregular beats by interpolation from other physiological signals. Frequency domain indices were computed from spectral analysis after cubic interpolation of the equidistant waveform and 4 Hz resampling.

Participants were asked to take 5 breaths into a calibration tube (completely filling and then emptying the bag of air) to calibrate respiratory volume. ECG, blood pressure, and respiration data were then recorded during three 5-min tasks. Baseline: A rectangle presented in the center of the TV screen changed color every 10 seconds and participants silently counted the number of blue rectangles (69). Sham Breathing: Participants breathed at a rate of ~14 breaths/min following a visual pacer (E-Z Air, Biofeedback Foundation of Europe, Montreal, Canada). They inhaled as the pacer moved vertically up and exhaled as it moved down. Resonance Breathing: Participants breathed at a rate of 6 breaths/min following the visual pacer to inhale as the pacer moved up and exhale as it moved down.

After completion of the physiological recording, participants were randomized into the eRPB or sham breathing group. Participants were given an iPhone programmed with CameraHRV [© Marco Altini, Amsterdam, Netherlands], an app that uses PPG in combination with a breathing pacer. Participants in both groups were instructed on how to open the app, enter the reason that prompted their app use, place their index finger over the camera lens to capture pulse data, and follow the app's pacer (inhaling as the vertical breathing bar moved up and exhaling as it moved down). The pacer was preset at 6 breaths per minutes for the eRPB group and 14 breaths per minute for the sham group. Randomization was double blind; iPhone app programming (eRPB vs. sham) was conducted by one unblinded researcher. Participants were asked to use their app for 5 min any time they anticipated or experienced a trigger and/or any other reason that might encourage them to drink or use drugs. In the event no such situations were encountered, participants were asked to use the app at the end of the day for 5 min. Preliminary observations of the PPG data suggested that participants breathed at the frequencies assigned to them during their self-initiated episodes of app use.

### Intervention

Participants were engaged in the intervention phase of the study for 8 weeks. The primary variables for the current analyses were collected during this phase. Research personnel met with participants weekly to upload app use data and collect self-report measures. Participants completed four Visual Analog Scale (VAS) questions to assess how much they were triggered by positive, negative, and temptation cues (per the IDTS cue categorization), and their perceived usefulness of the app. Participants also completed the Positive And Negative Affect Scale (PANAS) (70), PACS, and TLFB since last visit.

### Statistical approach

Analyses were conducted using SAS 9.4 (Cary, NC: SAS Institute Inc). Demographic and substance use comparisons of the eRPB and sham groups were conducted using ANOVA and chi-square.

**Figure 3:**
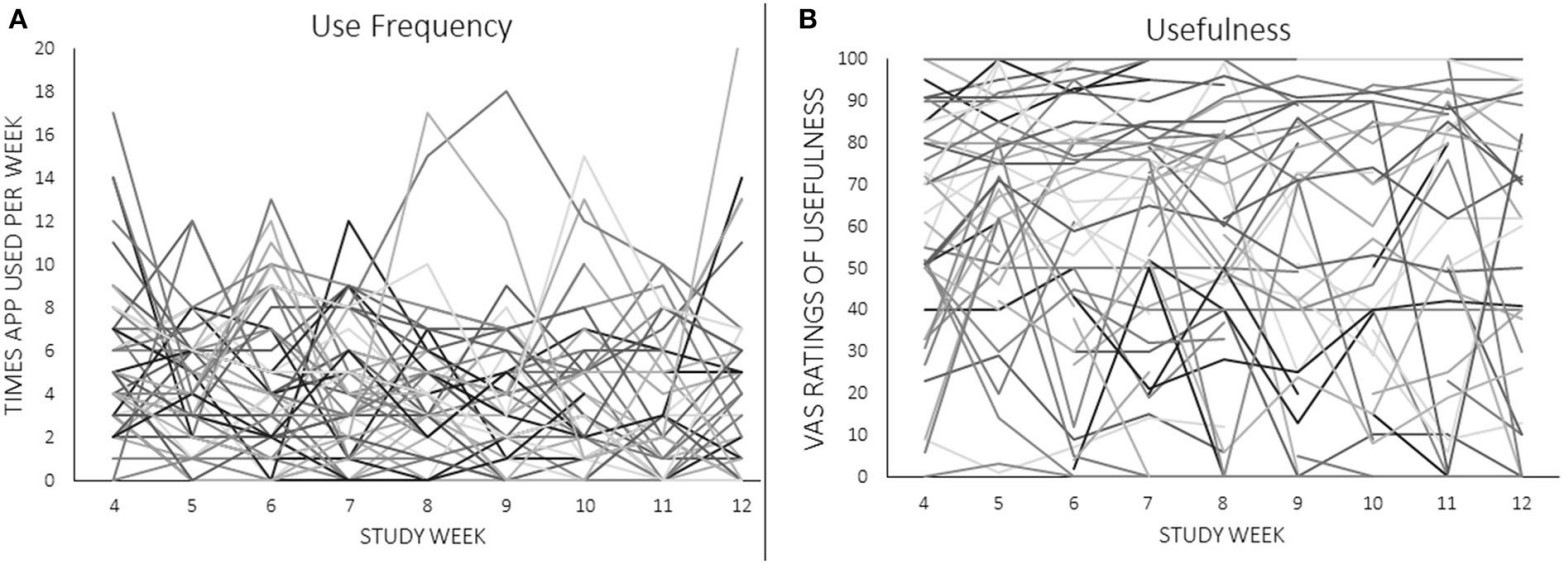
**(A,B)** Individual trajectories of app use frequency and ratings of usefulness across the 8-week intervention. Raw values of VAS ratings are shown.

Two measures of eRPB utility served as dependent variables in marginal means models: number of app uses per week as recorded from the iPhone app and weekly VAS ratings of usefulness. Traditional Intent-to-Treat analyses were not performed due to the seven participants with no outcome data points. Instead, maximum likelihood estimation was used to retain participants who were missing some, but not all, outcome data. Figure 3 depicts the week-to-week changes in both dependent variables, demonstrating a high degree of within- and between-subjects variability to be explained. Fixed predictors were extracted from the screening session [age, race (1 = Black, 0 = not Black), and frequency of exercise (weekly, monthly, or not at all)], the NJSAMS interview (existence of a current chronic medical condition), the SCID (lifetime AUD and SUD diagnoses), and the pre-intervention assessment [depressive and anxiety symptoms (BDI, BAI), cardiovascular parameters]. Cardiovascular predictors included mean power of high frequency (HF) HRV (0.15–0.40 Hz) during the baseline task, an index of resting parasympathetic activity, κ and peak power achieved at 0.1 Hz during resonance breathing, a proxy for baroreflex activity. Weekly VAS assessment of positive, negative, and temptation triggers during the intervention phase were included as time-varying predictors.

VAS questionnaires were scaled 0–100. Arcsin square root transformation $2^{*}\left(\sqrt{p}\right)$), was applied to all four VAS scales (Positive, Negative, and Temptation Triggers, Usefulness); this transform is a variance standardizing procedure for percentage (p) data (71). The natural log was taken of all physiological variables as is standard practice. Multicollinearity was assessed by first examining the bivariate correlation matrix and flagging correlations *r* ≈> 0.70 (72). Second, the specified dependent and independent variables, including interactions with condition randomization, were initially run in an ordinary least squares (OLS) regression model to examine the tolerances and variance inflation factors (VIF) for each entry in the regression equation.

Marginal means models were fit with a forward stepwise model selection procedure informed by fit indices (−2 log likelihood). Predictors that did not significantly improve model fit (non-significant X^2^) were dropped from the model. Model fit comparisons can be seen in the Table 1 noting which predictors were retained and which predictors were dropped from each model. All predictors were entered individually. Fixed demographic and health variables were entered first (age, race, exercise, medical condition), followed by substance use and affect variables (AUD/SUD, BDI, BAI), physiological indices (resting HF HRV, peak 0.1 Hz during resonance breathing), and then time-varying covariates (weekly negative, positive, and temptation triggers). The two dependent variables were hypothesized to be highly related and predictive of each other (i.e., frequency of use would be influenced by perceived usefulness; perceived usefulness would be influenced by the frequency of use). Thus, each dependent variable was entered into the opposing model as a time-varying predictor. Condition (eRPB vs. sham) and its interaction with all variables that remained in the model were then entered in the same manner. Robust standard errors were specified. Statistically significant effects (α = 0.05) are reported in-text with raw regression coefficients, *t*-statistics, and *p*-values.

**Table 1A:** Model fit statistics (compared to previous row): App use frequency.

	**-2LL**	**X^2^**	**df**	***p*** **-value**	**Residual**	***R*** **^2^ within**	**Dropped/Retained**
Null	2236.58	–	–	–	12.03	–	
Age	2230.26	6.32	1	0.012	11.85	0.015	R
Exercise	2213.75	16.51	1	<.001	11.39	0.0532	R
Medical condition	2210.75	3	1	0.083	11.31	0.0599	D
Race	2213.73	0.02	1	0.888	11.39	0.0532	D
BDI	2212.68	1.07	1	0.301	11.36	0.0557	D
BAI	2212.48	1.27	1	0.259	11.36	0.0557	D
AUD/SUD	2200.19	13.56	1	<0.001	11.03	0.0831	R
Resting HF HRV	2199.79	0.4	1	0.527	11.02	0.0840	D
Peak 0.1 Hz HRV	1991.8	207.99	1	<.001	8.3	0.3101	R
Negative Triggers	1634.95	356.85	1	<.001	8.69	0.2776	R
Positive Triggers	1515.58	119.37	1	<.001	8.29	0.3109	R
Temptation Triggers	1470.63	44.95	1	<.001	8.42	0.3001	R
App Usefulness	1388.6	82.03	1	<.001	7.27	0.3957	R
Condition X Age	1388.19	0.41	2	0.815	7.27	0.3957	D
Condition X Exercise	1388.37	0.23	2	0.891	7.26	0.3965	D
^**^Condition X AUD/SUD	**1379.89**	**8.71**	**2**	**0.013**	**7.05**	**0.4140**	**R**

Predictors with poor model fit (p > 0.05) were excluded from the model. Shaded rows indicate predictors retained in the final model.

** Final model (Use = Age, Exercise, AUD/SUD, peak 0.01 Hz HRV, Negative, Positive, Temptation, Usefulness, Condition, Condition*AUD/SUD).

**Table 1B:** Model fit statistics (compared to previous row): Usefulness.

	**-2LL**	**X^2^**	**df**	***p*** **-value**	**Residual**	***R*** **^2^ within**	**Dropped/Retained**
Null	1007.01	–	–	–	0.683		
Age	1006.35	0.66	1	0.41	0.682	0.0015	D
Exercise	1000.61	6.4	1	0.011	0.672	0.0161	R
Medical Condition	997.15	3.46	1	0.063	0.666	0.0249	D
Race	992.49	4.66	1	0.031	0.659	0.0351	R
BDI	992.42	0.07	1	0.791	0.659	0.0351	D
BAI	992.45	0.04	1	0.841	0.659	0.0351	D
AUD/SUD	974.89	17.6	1	<0.001	0.631	0.0761	R
Resting HF HRV	957.72	17.17	1	<0.001	0.605	0.1142	R
Peak 0.1 HZ HRV	891.78	65.94	1	<0.001	0.576	0.1567	R
Negative Triggers	810.52	81.26	1	<0.001	0.491	0.2811	R
Positive Triggers	752.12	58.4	1	<0.001	0.484	0.2914	R
Temptation Triggers	719.22	32.9	1	<0.001	0.477	0.3016	R
App Use Frequency	556.6	162.6	1	<0.001	0.405	0.4070	R
Condition X Exercise	544.93	11.67	2	0.003	0.388	0.4319	R
Condition X Race	541.8	3.13	1	0.077	0.383	0.4392	D
Condition X AUD/SUD	527.36	14.44	1	<0.001	0.365	0.4656	R
Condition XHF HRV	524.76	2.6	1	0.11	0.362	0.4700	D
Condition X Peak 0.1 Hz HRV	526.16	1.2	1	0.273	0.364	0.4671	D
Condition X Negative Triggers	519.97	7.39	1	0.007	0.356	0.4788	R
Condition X Positive Triggers	515.6	4.37	1	0.037	0.351	0.4861	R
Condition X Temptation Triggers	515.23	0.37	1	0.543	0.35	0.4876	D
^**^Condition X App Use Freq.	**505.5**	**10.1**	**1**	**<0.001**	**0.339**	**0.5037**	**R**

Predictors with poor model fit (p > 0.05) are removed from the model. Shaded rows indicate predictors retained in the final model.

** Final Model (Usefulness= Exercise, Race, HF HRV, Peak 0.01 Hz HRV, Negative, Positive, Temptation, Use Frequency, Condition, Condition*Exercise, Condition*AUD/SUD, Condition*Negative, Condition*Positive, Condition*Use Frequency).

## Results

### Multicollinearity

The bivariate correlation matrix can be seen in Table 2. A number of predictors were modestly associated with one another (*r* < 0.5). Scores on the BDI and BAI were correlated (*r* = 0.69) as were mean VAS trigger scales (Negative, Positive, Temptation, *r* = 0.32–0.58). VIF of main and interactive effects were well-below 10 and ranged from 1.55 to 8.71 [with the exception of condition, which was included in the calculation of interaction terms and thus had high collinearity with each interaction predictor (VIF≤ 16.63)]. All planned predictors were maintained in the analyses.

**Table 2:** Demographics and bivariate correlation matrix.

	**Mean/N**	**Var**	**1**	**2**	**3**	**4**	**5**	**6**	**7**	**8**	**9**	**10**	**11**	**12**	**13**
1. Condition			1	0.17	−0.21	−0.02	0.10	0.02	0.03	−0.24	0.10	0.01	0.13	−0.13	0.06
2. Age	33.5	8.51		1.00	0.01	−0.27 ^*^	0.13	−0.05	−0.24	−0.21	−0.19	−0.33	−0.31^*^	−0.33^*^	−0.31^*^
3. Race (Black)	12	—			1.00	−0.18	−0.07	−0.12	−0.15	−0.03	−0.32^*^	−0.32^*^	−0.35^*^	0.05	0.08
4. Exercise frequency (none/monthly/weekly)	19/13/25	—				1.00	−0.20	−0.01	−0.03	0.05	−0.10	0.15	0.12	0.09	0.25
5. Existing Medical condition	12	—					1.00	−0.01	0.14	−0.02	−0.14	0.30^*^	−0.14	−0.12	−0.13
6. BDI	14.97	10.37						1.00	0.69^*^	0.29^*^	0.44^*^	0.33^*^	0.40^*^	0.04	−0.16
7. BAI	16.92	12.63							1.00	0.12	0.29^*^	0.36^*^	0.29^*^	0.01	−0.16
8. AUD/SUD/ASUD	11/18/28	–								1.00	0.18	0.19	0.14	0.18	0.02
9. Negative Triggers	1.29	0.689									1.00	0.32^*^	0.58^*^	0.00	0.01
10. Positive Triggers	0.848	0.618										1.00	0.53^*^	0.02	−0.01
11. Temptation Triggers (Arcsin sqrt)	0.99	0.636											1.00	0.09	0.12
12. High frequency HRV	5.78	1.52												1.00	0.59^*^
13. Peak 0.1 Hz HRV	12.75	1.12													1.00

* *p* < 0.05.

BDI, Beck Depressive Inventory; BAI, Beck Anxiety Inventory; AUD, Alcohol Use Disorder; SUD, Substance Use Disorder; ASUD, comorbid Alcohol and Substance Use Disorder; Arcsin squareroot transformation was applied to Negative, Positive, and Temptation Trigger values; High frequency and Peak 0.1 HZ HRV measures are log transformed.

### Frequency of app use

A summary of significant findings can be seen in Table 3. Two baseline characteristics were associated with app use frequency. More frequent use was significantly associated with having an AUD diagnosis (ß = 2.41, t35 = 2.32, *p* = 0.026) and associated at a trend level with a higher peak 0.1 Hz index during resonance breathing (ß = 0.568, t35 = 1.75, *p* = 0.088). Of the time-varying covariates, more frequent use was associated with fewer weekly exposures to positive triggers (ß = −1.11, t238 = −3.46, *p* < 0.001) but high weekly ratings of app usefulness (ß = 1.35, t238 = 3.46, *p* < 0.001). The residual *r*^2^ determined that the final model accounted for 41% of the within-subject variance.

**Table 3:** Standardized beta weights of significant predictors.

	**Frequency of use**		**Usefulness**	
**Baseline characteristic predictors**	**ß**	***p*** **-value**	**ß**	***p*** **-value**
AUD diagnosis	+2.41	0.026		
Peak 0.1 Hz during resonance	+0.568	0.088		
High frequency HRV			−0.15	0.05
**Time-varying predictors**				
Positive trigger exposure	−1.11	<0.001		
Negative trigger exposure			+0.508	<0.001
^*^Frequency of use X condition interaction			+0.830	0.045
Usefulness	+1.35	<0.001		

ß = Standardized beta weights.

* Frequency of use was only a significant predictor of Usefulness in the eRPB group.

### Ratings of app usefulness

**Figure 4:**
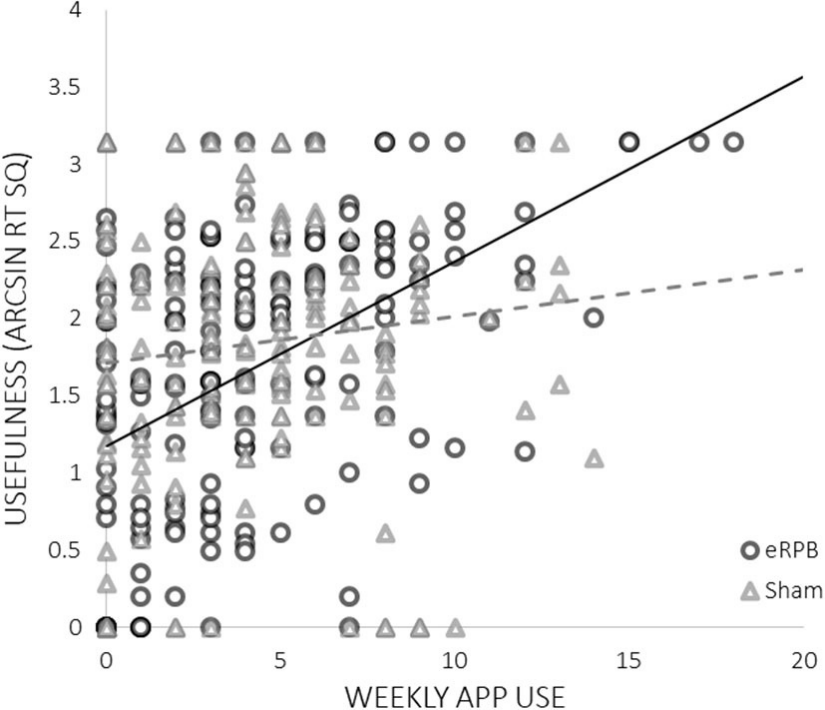
Relationship between app use and ratings of usefulness by condition.

High ratings of usefulness were associated with both lower basal high frequency HRV at intake (ß = −0.150, t33 = −2.01, *p* = 0.05) and greater weekly exposure to negative triggers (ß = 0.508, *t*(241) = 4.48, *p* < 0.001). There was a significant interaction between frequency of use and condition; more frequent use was predictive of higher ratings of usefulness only in the eRPB group (Figure 4, ß = 0.830, *t*(241) = 2.01, *p* = 0.045). The residual *r*^2^ suggested that the final model accounted for 50% of the within-subject variance.

## Discussion

The accessibility of eHealth platforms is a powerful advancement for the delivery of just-in-time interventions to potentially reduce the influence of daily life triggers on substance use behavior. This study sought to identify factors that affect compliance with the use of an app-based breathing intervention as well as its perceived usefulness in dampening alcohol and drug craving in-the-moment in women receiving outpatient treatment for SUD. Identifying individuals who may be most likely to use app-based interventions can contribute to efficient screening (i.e., person-intervention matching) and support aftercare planning that best promotes long term recovery. Further, triangulation of app use with its perceived usefulness can provide information about internal and contextual moderators of eRBP's effectiveness for craving blockade.

The results of the current analyses supplement our prior demonstration of the efficacy of eRPB as an anti-craving, just-in-time, bottom-up intervention. They provide several notable observations about who engaged in the study paradigm and for whom it was most useful. Perhaps most compelling was the observation that participants in the eRPB group, but not the sham breathing group, who used the app more frequently rated the app as most useful for countering triggers of substance craving. The sham control condition had high face validity and paced respiration within the lower range of normal respiration rates. That the association between use and usefulness was limited to the eRPB group supports the study's hypothesis that it is the activation of the HR baroreflex system, as opposed to a general calming or distraction effect of paced breathing, that dampens arousal and stabilizes craving.

Beyond this support for eRPB's ability to activate putative physiologic mechanisms of behavior change, the present results suggest that a number of state-level physiologic factors influence acceptability and usefulness of the app in the sample as a whole. Women who entered the intervention with lower resting parasympathetic activity found the app to be more useful. This makes intuitive sense from a physiological perspective as women with signs of autonomic dysregulation would be most likely to benefit from an intervention aimed at modulating autonomic arousal, and is also consistent with our previous study that showed lower basal HRV was a predictor of HRV biofeedback efficacy (39). There was also a trend for women who generated larger responses to eRPB during their initial laboratory visit to use the app more frequently; again, this makes intuitive physiological sense in that when an app accomplishes what it sets out to do (in this case, maximizing heart rate oscillations), it is more likely to be used. Several factors, however, can influence the 0.1 Hz HRV peak obtained during eRPB (45, 73); thus, more research with larger samples is needed to discern the effects of sex, race, and ethnicity. Larger samples may further identify momentary factors related to the intervention's efficacy. Nonetheless, this study found evidence of pre-intervention physiological predictors of app utility, suggesting that simple, brief cardiovascular assessments at intake could help identify those who are mostly likely to benefit from an eRPB add-on to treatment to mitigate craving and support long-term recovery.

Importantly, the strongest predictors in both models were contextual in nature. Weeks of high exposure to negative triggers were associated with high ratings of usefulness. In contrast, weeks with high exposure to positive triggers were associated with less frequent use. eRPB is designed as a just-in-time intervention that is non-invasive and easy to perform in the context of daily life, without expensive equipment or the need for clinical supervision. The identification of time-varying predictors of frequency of app use and self-reported ratings of app utility supports eRPB as such an intervention. Furthermore, considered in the context of our prior studies linking eRPB and HRV biofeedback to craving modulation (39, 48, 55, 74) and the widely held perspective of craving as a highly contextual state (as opposed to trait) factor (75, 76), it is perhaps unsurprising that the greatest predictors of app use and usefulness are the equally dynamic daily life triggers that a person navigates during recovery.

A previous study of a traditional, clinician-led course of HRV biofeedback in a college recovery sample found that within-person variations in depression symptoms (i.e., weeks when symptoms were higher than the person's average level) significantly contributed to the prediction of craving during an 8-week intervention (74). We thus hypothesized that mood would influence how useful the app would be in the face of craving, or how frequently participants would use it. However, we found no relationship between depression symptoms and participants' frequency of use or reported usefulness of the resonance breathing app. A likely explanation for the difference is that mood is a similarly dynamic state that fluctuates in tandem with craving. Alayan and colleagues found a significant effect of within-person variability in weekly BDI, but not mean-level between-subjects BDI, on craving. The current study assessed BDI at the initial assessment, and not continuously throughout the intervention. Regular monitoring of mood states in conjunction with craving may therefore be necessary to clarify times when the resonance breathing app may be most beneficial.

Control of craving is an important but complex intervention target. Biobehavioral interventions such as eRPB may be valuable as components of a comprehensive treatment regimen along with pharmacological and other behavioral interventions. Currently approved anti-craving medications, primarily naltrexone and acamprosate, have demonstrated therapeutic benefit (77, 78). Off-label use of anticonvulsants (topiramate, gabapentin) and atypical antipsychotics (aripiprazole) also appear to decrease craving (25, 26, 79). Although anti-craving medications remain under-prescribed, evidence suggests that they are mostly like to be effective when used in combination with psychological treatments (80); eRPB can be seamlessly integrated with pharmacological treatment to augment craving control.

### Limitations and future directions

One *a priori* predictor was not included in analyses or reported here. Upon launching the resonance breathing app, participants were prompted to enter the reason for use. Our intention was to code and categorize entries; however, women had difficulty naming their reasons for app use in the moment and the open text format of the prompts was not successful. It may be that the craving hyperarousal state sufficiently blocked cognitive processes and impeded participants' capacity to generate a free-response answer.

During the weekly in-person assessments, clinical researchers probed whether participants had difficulty using the breathing app, but responses were not recorded because this information primarily was used to solve technical issues. However, such qualitative data about the app's utility could help identify barriers to using the eHealth intervention. Such information is integral to dissemination and implementation and should be considered in future large scale trials.

The just-in-time self-administration aspect of the breathing app was a key component of the clinical trial. While women in the eRPB group who used the app more frequently found the app to be more useful, the mean number of uses per week was 4 (range 0–21), despite study instructions of at least daily use. There were possibly times when participants experienced craving and did not use the app, as well as days when craving triggers were not encountered. Recent computational modeling approaches using in-the-field autonomic signals, GPS indicators of trigger-heavy environments, and ecological momentary assessment (EMA) of subjective stress successfully predicted substance craving with a high degree of accuracy (16, 17, 81). Development of a neurocardiac intervention that is able to “ping” an individual to launch the resonance breathing app based on EMA and continuous physiological and location data may improve upon the self-administration model. This is a fertile area for future research.

## Conclusion

eRPB, as a just-in-time, body-focused, anti-craving intervention has been shown effective in dampening craving. The ability to match patient phenotypes and momentary craving triggers with its utility optimizes use of continuously evolving intervention technology. These data provide implementation information to help identify treatment-seekers most suited for bottom-up arousal modulation. Brief assessment of baseline cardiovascular function and identification of momentary exposure to negative triggers may help target ideal candidates, and instances, of need to supplement treatment with resonance breathing.

## Data availability statement

The raw data supporting the conclusions of this article will be made available by the authors, without undue reservation.

## Ethics statement

The studies involving human participants were reviewed and approved by Rutgers University Arts and Science Institutional Review Board. The patients/participants provided their written informed consent to participate in this study.

## Author contributions

JP performed formal data analysis and drafted the current manuscript. MB was responsible for funding acquisition, conceptualization, study design, review and editing the current manuscript, and supervision. AP performed formal data analysis and contributed to writing of the manuscript. SU, ST, and JM were responsible for conducting the study procedures, data management, preliminary analyses, and drafting an earlier version of this work. JB was responsible for funding acquisition, conceptualization, study design, review and editing the current manuscript, and supervision. All authors contributed to the article and approved the submitted version.

## Funding

Project IMPACT was funded by the National Institute of Health (R01AA023667, K02AA025123, K24AA021778). JP was supported by the Molecular Neuroscience of Drug Abuse Research Training grant (T32AA028254) NIAAA had no role in the study design, collection, analysis, or interpretation of the data, writing the manuscript, or deciding to submit the paper for publication.

## Conflict of interest

The authors declare that the research was conducted in the absence of any commercial or financial relationships that could be construed as a potential conflict of interest.

## Publisher's note

All claims expressed in this article are solely those of the authors and do not necessarily represent those of their affiliated organizations, or those of the publisher, the editors and the reviewers. Any product that may be evaluated in this article, or claim that may be made by its manufacturer, is not guaranteed or endorsed by the publisher.

## References

[1] Baker TB Piper ME McCarthy DE Majeskie MR Fiore MC. Addiction motivation reformulated: an affective processing model of negative reinforcement. Psychol Rev. (2004) 111:33–51. 10.1037/0033-295X.111.1.33 14756584

[2] Tiffany ST Wray J. The continuing conundrum of craving. Addiction. (2009) 104:1618–9. 10.1111/j.1360-0443.2009.02588.x 19558571

[3] Brewer JA Elwafi HM Davis JH. Craving to quit: Psychological models and neurobiological mechanisms of mindfulness training as treatment for addictions. Psychol Add Behav: J Soc Psychol Addict Behav. (2013) 27:366–79. 10.1037/a0028490 22642859 PMC3434285

[4] Fatseas M Serre F Alexandre JM Debrabant R Auriacombe M Swendsen J. Craving and substance use among patients with alcohol, tobacco, cannabis or heroin addiction: a comparison of substance- and person-specific cues. Addiction. (2015) 110:1035–42. 10.1111/add.12882 25688760

[5] Field M Jones A. Elevated alcohol consumption following alcohol cue exposure is partially mediated by reduced inhibitory control and increased craving. Psychopharmacology. (2017) 234:2979–88. 10.1007/s00213-017-4694-6 28741032 PMC5591800

[6] Rosenberg H. Clinical and laboratory assessment of the subjective experience of drug craving. Clin Psychol Rev. (2009) 29:519–34. 10.1016/j.cpr.06002 19577831

[7] Weiss F. Neurobiology of craving, conditioned reward, and relapse. Curr Opin Pharmacoly. (2005) 5:9–19. 10.1016/j.coph.11001 15661620

[8] Foltin RW Haney M. Conditioned effects of environmental stimuli paired with smoked cocaine in humans. Psychopharmacology. (2000) 149:24–33. 10.1007/s002139900340 10789879

[9] Sinha R Talih M Malison R Cooney N Anderson GM Kreek MJ. Hypothalamic-pituitary-adrenal axis and sympatho-adreno-medullary responses during stress-induced and drug cue-induced cocaine craving states. Psychopharmacology. (2003) 170:62–72. 10.1007/s00213-003-1525-8 12845411

[10] Back SE Gros DF McCauley JL Flanagan JC Cox E Barth KS. Laboratory-induced cue reactivity among individuals with prescription opioid dependence. Add Behav. (2014) 39:1217–23. 10.1016/j.addbeh.04007 24813546 PMC4059191

[11] Culbertson C Nicolas S Zaharovits I London ED La Garza Richard De Brody AL Newton TF. Methamphetamine craving induced in an online virtual reality environment. Pharmacol Biochem Behav. (2010) 96:454–60. 10.1016/j.pbb.07005 20643158 PMC2951480

[12] Ingjaldsson JT Laberg JC Thayer JF. Reduced heart rate variability in chronic alcohol abuse: Relationship with negative mood, chronic thought suppression, and compulsive drinking. Biol Psychiatry. (2003) 54:1427–36. 10.1016/S0006-3223(02)01926-1 14675808

[13] Dhawan A Kumar R Yadav S Tripathi BM. The enigma of craving. Indian J Psychiatry. (2002) 44:138–43. 21206559 PMC2954341

[14] Ooteman W Koeter MWJ Vserheul R Schippers GM Brink W van den. Measuring craving: an attempt to connect subjective craving with cue reactivity. Alcohol: Clin Exp Res. (2006) 30:57–69. 10.1111/j.1530-0277.2006.00019.x 16433732

[15] Heishman SJ Lee DC Taylor RC Singleton EG. Prolonged duration of craving, mood, and autonomic responses elicited by cues and imagery in smokers: effects of tobacco deprivation and sex. Exp Clin Psychopharmacol. (2010) 18:245–56. 10.1037/a0019401 20545389 PMC2896221

[16] Panlilio LV Stull SW Bertz JW Burgess-Hull AJ Lanza ST Curtis BL. Beyond abstinence and relapse II: momentary relationships between stress, craving, and lapse within clusters of patients with similar patterns of drug use. Psychopharmacology. (2021) 238:1513–29. 10.1007/s00213-021-05782-2 33558983 PMC8141007

[17] Preston KL Kowalczyk WJ Phillips KA Jobes ML Vahabzadeh M Lin JL. Before and after: craving, mood, and background stress in the hours surrounding drug use and stressful events in patients with opioid-use disorder. Psychopharmacology. (2018) 235:2713–23. 10.1007/s00213-018-4966-9 29980821 PMC6119104

[18] Franklin TR Wang Z Wang J Sciortino N Harper D Li Y. Limbic activation to cigarette smoking cues independent of nicotine withdrawal: a perfusion fMRI study. Neuropsychopharmacology. (2007) 32:2301–9. 10.1038/sj.npp.1301371 17375140

[19] Kühn S Gallinat J. Common biology of craving across legal and illegal drugs – a quantitative meta-analysis of cue-reactivity brain response. Eu J Neurosci. (2011) 33:1318–26. 10.1111/j.1460-9568.2010.07590.x 21261758

[20] Seo D Lacadie CM Tuit K Hong KI Constable RT Sinha R. Disrupted ventromedial prefrontal function, alcohol craving, and subsequent relapse risk. JAMA Psychiatry. (2013) 70:727–39. 10.1001/jamapsychiatry.2013.762 23636842 PMC3788824

[21] Oslin DW Cary M Slaymaker V Colleran C Blow FC. Daily ratings measures of alcohol craving during an inpatient stay define subtypes of alcohol addiction that predict subsequent risk for resumption of drinking. (2009). Drug Alcohol Depend. 103:131–6. 10.1016/j.drugalcdep.03009 19443131 PMC12272362

[22] Paliwal P Hyman SM Sinha R. Craving predicts time to cocaine relapse: further validation of the now and brief versions of the cocaine craving questionnaire. Drug Alcohol Depend. (2008) 93:252–9. 10.1016/j.drugalcdep.10002 18063320 PMC2254317

[23] Boettiger C Chanon VW Kelm MK. Brain mechanisms of addiction treatment effects. in Biological Research on Addiction: Comprehensive Addictive Behaviors and Disorders. Academic Press (2013) 2:431–440.

[24] Haass-Koffler CL Goodyear K Zywiak WH Leggio L Kenna GA Swift RM. Comparing and combining topiramate and aripiprazole on alcohol-related outcomes in a human laboratory study. Alcohol Alcoholism. (2018) 53:268–76. 10.1093/alcalc/agx108 29281033 PMC5913672

[25] Haass-Koffler CL Swift RM Leggio L. Noradrenergic targets for the treatment of alcohol use disorder. Psychopharmacology. (2018) 235:1625–34. 10.1007/s00213-018-4843-6 29460163 PMC5995154

[26] Martinotti G Orsolini L Fornaro M Vecchiotti R Berardis De Iasevoli D. Aripiprazole for relapse prevention and craving in alcohol use disorder: Current evidence and future perspectives. Expert Opinion Investigat Drugs. (2016) 25:719–28. 10.1080/13543784.2016.1175431 27098451

[27] Rösner S Hackl-Herrwerth A Leucht S Lehert P Vecchi S Soyka M. Acamprosate for alcohol dependence. Cochrane Database Syst Rev. (2010) 9:CD004332. 10.1002/14651858.CD004332.pub2 20824837 PMC12147086

[28] Bates ME Price JL Buckman JF. Neuropsychological and Biological Influences on Drinking Behavior Change. In J. A. Tucker and K. Witkiewitz (Eds.), Dynamic Pathways to Recovery from Alcohol Use Disorder: Meaning and Methods. Cambridge University Press. (2022) (pp. 60–76). 10.1017./9781108976213.008

[29] Eddie D Bates ME Buckman JF. Closing the brain–heart loop: Towards more holistic models of addiction and addiction recovery. Addict Biol. (2020) 3:12958. 10.1111./adb.12958 32783345 PMC7878572

[30] Eddie D Price JL Bates ME Buckman JF. Substance use and addiction affect more than the brain: the promise of neurocardiac interventions. Curr Addict Rep. (2021) 8:431–9. 10.1007/s40429-021-00379-3 35449896 PMC9017547

[31] Hartwell KJ Johnson KA Li X Myrick H LeMatty T George MS. Neural correlates of craving and resisting craving for tobacco in nicotine dependent smokers. Add Biol. (2011), 16:654–66. 10.1111/j.1369-201100340.x 21790899 PMC3182826

[32] Wang GB Zhang XL Zhao LY Sun LL Wu P Lu L. Drug-related cues exacerbate decision making and increase craving in heroin addicts at different abstinence times. Psychopharmacology. (2012) 221:701–8. 10.1007/s00213-011-2617-5 22207241

[33] Lehrer P. How does heart rate variability biofeedback work? Resonance, the baroreflex, and other mechanisms. Biofeedback. (2013) 41:26–31. 10.5298/1081-5937-41.1.0225101026

[34] Vaschillo EG Vaschillo B Lehrer PM. Characteristics of resonance in heart rate variability stimulated by biofeedback. Appl Psychophysiol Biofeedback. (2006) 31:129–42. 10.1007/s10484-006-9009-3 16838124

[35] Vaschillo EG Vaschillo B Buckman JF Pandina RJ Bates ME. The investigation and clinical significance of resonance in the heart rate and vascular tone baroreflexes. In Fred A, Filipe J, Gamboa H (Eds.), Biomedical Engineering Systems and Technologies. (2011). Springer Berlin Heidelberg (pp. 224–237).

[36] Lehrer PM Vaschillo E Vaschillo B. Resonant frequency biofeedback training to increase cardiac variability: rationale and manual for training. Appl Psychophysiol Biofeedback. (2000) 25:177–91. 10.1023/A:1009554825745 10999236

[37] Crestani CC Tavares RF Alves FHF Resstel LBM Correa FMA. Effect of acute restraint stress on the tachycardiac and bradycardiac responses of the baroreflex in rats. Stress. (2010) 13:61–72. 10.3109/10253890902927950 20105054

[38] Norcliffe-Kaufmann L. Stress and the baroreflex. Autonomic Neurosci. (2022) 238:102946. 10.1016/j.autneu.2022.102946 35086020

[39] Eddie D Kim C Lehrer P Deneke E Bates ME. A pilot study of brief heart rate variability biofeedback to reduce craving in young adult men receiving inpatient treatment for substance use disorders. Appl Psychophysiol Biofeedback. (2014) 39:181–92. 10.1007/s10484-014-9251-z 25179673 PMC4221295

[40] Mun EY von Eye A Bates ME Vaschillo EG. Finding groups using model-based cluster analysis: heterogeneous emotional self-regulatory processes and heavy alcohol use risk. Develop Psychol. (2008) 44:481–95. 10.1037/0012-442.481 18331138 PMC2909466

[41] Udo T Bates ME Mun EY Vaschillo EG Vaschillo B Lehrer P. Gender Differences in acute alcohol effects on self-regulation of arousal in response to emotional and alcohol-related picture cues. Psychol Addict Behav : J Soc Psychol Addict Behav. (2009) 23:196–204. 10.1037/a0015015 19586136 PMC2964059

[42] Benarroch EE. The central autonomic network: functional organization, dysfunction, and perspective. Mayo Clinic Proceedings. (1993) 68:988–1001. 10.1016/S0025-6196(12)62272-1 8412366

[43] Bates ME Lesnewich LM Uhouse SG Gohel S Buckman JF. Resonance-paced breathing alters neural response to visual cues: proof-of-concept for a neuroscience-informed adjunct to addiction treatments. Front Psych. (2019) 10:624. 10.3389./fpsyt.2019.00624 31543840 PMC6739688

[44] Hinterberger T Walter N Doliwa C Loew T. The brain's resonance with breathing—Decelerated breathing synchronizes heart rate and slow cortical potentials. J Breath Res. (2019) 13:046003. 10.1088/1752-7163/ab20b2 31071704

[45] Lehrer PM Vaschillo EG Vidali V. Heart rate and breathing are not always in phase during resonance frequency breathing. Appl Psychophysiol Biofeedback. (2020) 45:145–52. 10.1007/s10484-020-09459-y 32285231

[46] Lin IM Wang SY Fan SY Peper E Chen SP Huang CY. a single session of heart rate variability biofeedback produced greater increases in heart rate variability than autogenic training. Appl Psychophysiol Biofeed. (2020) 45:343–50. 10.1007/s10484-020-09483-y 32767160

[47] Yen CF Ko CH Hsu CY Wu HC Yang YY Wang PW. A pilot randomized control study on effect brief heart rate variability biofeedback as a complementary treatment in men with methamphetamine use disorder. Int J Environ Res Public Health. (2022) 19:5230. 10.3390/ijerph19095230 35564623 PMC9105208

[48] Alayan N Eller L Bates ME Carmody DP. Current evidence on heart rate variability biofeedback as a complementary anticraving intervention. J Alternat Complement Med. (2018) 24:1039–50. 10.1089/acm.2018.0019 29782180 PMC6422009

[49] Zaccaro A Piarulli A Laurino M Garbella E Menicucci D Neri B. How breath-control can change your life: a systematic review on psycho-physiological correlates of slow breathing. Front Human. (2018) 12:353. 10.3389./fnhum.2018.00353 30245619 PMC6137615

[50] Sarker H Sharmin M Ali AA Rahman M Bari M Hossain R Kumar S. Assessing the availability of users to engage in just-in-time intervention in the natural environment. Proceed 2014 ACM Intl Joint Conf Pervasive Ubiquitous Comp. (2014) 8:909–920. 10.1145./2632048.2636082 25798455 PMC4365928

[51] Yanez B McGinty HL Mohr DC Begale MJ Dahn JR Flury SC. Feasibility, acceptability, and preliminary efficacy of a technology-assisted psychosocial intervention for racially diverse men with advanced prostate cancer. Cancer. (2015) 121:4407–15. 10.1002/cncr.29658 26348661 PMC4670576

[52] Yeh VM Bergner EM Bruce MA Kripalani S Mitrani VB Ogunsola TA. Can precision medicine actually help people like me? African American and hispanic perspectives on the benefits and barriers of precision medicine. Ethn Dis. (2020) 30:149–58. 10.18865/ed.30.S1.149 32269456 PMC7138449

[53] Jang M Johnson CM D'Eramo-Melkus G Vorderstrasse AA. Participation of racial and ethnic minorities in technology-based interventions to self-manage type 2 diabetes: a scoping review. J Transcult Nurs. (2018) 29:292–307. 10.1177/1043659617723074 28826353

[54] Ramos G Chavira DA. Use of technology to provide mental health care for racial and ethnic minorities: evidence, promise, and challenges. Cognit Behav Pract. (2022) 29:15–40. 10.1016/j.cbpra.10004

[55] Price JL Bates ME Morgano J Todaro S Uhouse SG Vaschillo E. Effects of arousal modulation via resonance breathing on craving and affect in women with substance use disorder. Addict Behav. (2022) 127:107207. 10.1016/j.addbeh.2021.107207 34953433 PMC9069782

[56] ElGeili ESS Bashir TZ. High-risk relapse situations and self-efficacy: comparison between alcoholics and heroin addicts. Addict Behav. (2004) 29:753–758. 10.1016/j.addbeh.02003 15135557

[57] ElGeili ESS Bashir TZ. Precipitants of relapse among heroin addicts. Addict Disord Their Treat. (2005) 4:29–38.

[58] Abulseoud OA Karpyak VM Schneekloth T Hall-Flavin DK Loukianova LL Geske JR. A retrospective study of gender differences in depressive symptoms and risk of relapse in patients with alcohol dependence. Am J Addict. (2013) 22:437–42. 10.1111/j.1521-0391.2013.12021.x 23952888 PMC3748388

[59] Najavits LM. Seeking safety: an evidence-based model for substance abuse and trauma/PTSD. In Witkiewitz KA and Marlatt GA (Eds.), Therapist's Guide to Evidence-Based Relapse Prevention. Academic Press. (2007) (pp. 141–167). 10.1016/B978-012369429-4/50037-9

[60] Miller W Rollnick S. Motivational Interviewing: Preparing People to Change Addictive Behavior. New York, NY: Gulliford Press (1991).

[61] Lieberman AF Van horn P Ippen CG. Toward evidence-based treatment: child-parent psychotherapy with preschoolers exposed to marital violence. J Am Aca Child Adoles Psych. (2005) 44:1241–8. 10.1097/01.chi.0000185970258 16292115

[62] First MB Williams JBW Karg RS Spitzer RL. Structured Clinical Interview for DSM-5, Research Version. Structured Clinical Interview for DSM-5 Disorders, Research Version (SCID-5 for DSM-5, Research Version; SCID-5-RV). Arlington, VA: American Psychiatric Association (2015).

[63] Sheehan DV Lecrubier Y Harnett-Sheehan K Amorim P Janvas J Weiller E. The Mini International Neuropsychiatric Interview (M.I.N.I.): The Development Validation of a Structured Diagnostic Psychiatric Interview. J Clin Psychiatry. (1998) 59. Available online at: http://www.psychiatrist.com/JCP/article/Pages/1998/v59s20/v59s2005.aspx 9881538

[64] Annis HM Martin G. Inventory of Drug-Taking Situations. Toronto: Addiction Research Foundation (1985).

[65] Sobell LC Sobell MB. Timeline follow-back. In Litten RZ, Allen JP (Eds.), Measuring Alcohol Consumption: Psychosocial and Biochemical Methods. Humana Press. (1992) (pp. 41–72). 10.1007./978-1-4612-0357-5_3

[66] Flannery B Volpicelli JR Pettinati HM. Psychometric properties of the Penn alcohol craving scale. Alcohol: Clin Experim Res. (1999) 23:1289–95. 10470970

[67] Beck AT Ward CH Mendelson M Mock J Erbaugh J. An inventory for measuring depression. Arch Gen Psychiatry. (1961) 4:561–71. 13688369 10.1001/archpsyc.1961.01710120031004

[68] Beck AT Steer RA. Beck Anxiety Inventory Manual. San Antonio, TX., Psychological Corporation. (1993). Available online at: https://www.pearsonassessments.com/store/usassessments/en/Store/Professional-Assessments/Personality-%26-Biopsychosocial/Beck-Anxiety-Inventory/p/100000251.html

[69] Jennings JR Kamarck T Stewart C Eddy M Johnson P. Alternate cardiovascular baseline assessment techniques: Vanilla or resting baseline. Psychophysiology. (1992) 2:742–750. 10.1111/j.1469-8986.1992.tb02052.x 1461961

[70] Watson D Clark LA Tellegen A. Development and validation of brief measures of positive and negative affect: the PANAS scales. J Pers Soc Psychol. (1988) 54:1063. 10.1037/0022-3514.54.6.1063 3397865

[71] Sokal RR Rohlf FJ. Biometry. New York, NY: Macmillan (1995).

[72] Tabachnick BG Fidell LS. Using multivariate statistics (Seventh edition). Pearson. (2018).

[73] Heiss S Vaschillo B Vaschillo EG Timko CA Hormes JM. Heart rate variability as a biobehavioral marker of diverse psychopathologies: a review and argument for an “ideal range.” Neurosci Biobehav Rev. (2020) 121:144–55. 10.1016/j.neubiorev.12.004 33309905

[74] Alayan N Eddie D Eller L Bates ME Carmody DP. Substance craving changes in university students receiving heart rate variability biofeedback: a longitudinal multilevel modeling approach. Add Behav. (2019) 97:35–41. 10.1016/j.addbeh.05005 31132527 PMC7454170

[75] Drummond DC Litten RZ Lowman C Hunt WA. Craving research: future directions. Addiction. (2000) 95:247–55. 10.1046/j.1360-0443.95.8s2.13.x 11002919

[76] Tiffany ST. Cognitive concepts of craving. Alcohol Res Health. (1999) 23:215–24. 10890817 PMC6760370

[77] Calabrese J Brown J Hasan M Neuhut S Jo Y. Hospital readmission in alcohol use disorder patients: the role of anti-craving medications and discharge disposition. HCA Healthcare J Med. (2022) 3:1243. 10.36518/2689-0216.1243 PMC10324689 37426377

[78] Holzbach R Stammen G Kirchhof U Scherbaum N. The prescription of anticraving medication and its economic consequences. Eur Addict Res. (2019) 25:224–8. 10.1159/000500521 31216535

[79] Furieri FA Nakamura-Palacios EM. Gabapentin reduces alcohol consumption and craving: a randomized, double-blind, placebo-controlled trial. J Clin Psychiatry. (2007) 68:1691–700. 10.4088/JCP.v68n1108 18052562

[80] Mong J Ahamad K Bach P. Anticraving medication for moderate to severe alcohol use disorder. CMAJ. (2021) 193:E695–E695. 10.1503/cmaj.200895 33972223 PMC8157997

[81] Epstein DH Tyburski M Kowalczyk WJ Burgess-Hull AJ Phillips KA Curtis BL. Prediction of stress and drug craving ninety minutes in the future with passively collected GPS data. Npj Digital Medicine. (2020) 3:1–12. 10.1038/s41746-020-0234-6 32195362 PMC7055250

